# A study on the variability and correlation of ocular biological measurement parameters in adult myopic patients

**DOI:** 10.3389/fmed.2024.1526703

**Published:** 2025-01-07

**Authors:** Fangxing Zhou, Nan Chen, Hui Qian, Di Gong, Kunke Li

**Affiliations:** ^1^Fuzhou Aier Eye Hospital, Fuzhou, Jiangxi, China; ^2^Eye Hospital, Nanjing Medical University, Nanjing, Jiangsu, China; ^3^Shenzhen Eye Institute, Shenzhen Eye Hospital, Jinan University, Shenzhen, Guangdong, China

**Keywords:** myopia, axial length, spherical equivalent, corneal radius of curvature, axial length/corneal radius of curvature ratio

## Abstract

**Objective:**

This study aims to explore the differences in ocular parameters among adult myopic patients with different degrees of myopia and axial lengths, and to investigate the correlations between these ocular parameters.

**Methods:**

This single-center observational study collected clinical data from myopic patients aged 18–45 years who visited the Eye Hospital of Nanjing Medical University between January and June 2023. The data included laterality, diopter of spherical power (DS), diopter of cylindrical power (DC), spherical equivalent (SE), axial length (AL), central corneal thickness (CCT), flat meridian keratometry (K1), steep meridian keratometry (K2), mean keratometry (Km), anterior chamber depth (ACD), corneal radius of curvature (CRC), and axial length/corneal radius of curvature ratio (AL/CRC). Following predefined inclusion and exclusion criteria, 1,026 eyes were included in the study. Patients were grouped based on SE and AL parameters into different degrees of myopia. Analysis of variance (ANOVA) and Welch ANOVA were used to compare intergroup differences. Spearman correlation coefficients were calculated to analyze the correlations between parameters, and linear regression and ROC curve analyses were performed.

**Results:**

Significant differences (*p* < 0.05) were found among mild, moderate, and high myopia groups in parameters such as DS, DC, AL, K1, Km, ACD, CRC, and AL/CRC. Significant differences (*p* < 0.05) were also found in DS, DC, SE, CCT, K1, K2, Km, ACD, CRC, and AL/CRC among different axial length groups. Spearman correlation analysis showed a strong correlation between AL and DS, SE, and between AL/CRC and DS, SE, AL. Linear regression analysis revealed that the coefficient of determination (R^2^) for AL and SE was 0.699, and for AL/CRC and SE, it was 0.861. ROC curve analysis demonstrated high accuracy for both AL and AL/CRC in identifying high myopia, with an AUC of 0.952 for AL/CRC, which was superior to the AUC of 0.905 for AL (*p* < 0.05).

**Conclusion:**

This study found significant differences in ocular parameters among patients with different degrees of myopia and axial lengths. There was a significant negative correlation between AL, AL/CRC, and SE. Compared to AL, AL/CRC had a stronger correlation with SE and higher accuracy in identifying high myopia.

## 1 Introduction

Myopia, a common and complex refractive error, impairs the ability to see distant objects clearly and is often associated with severe complications such as glaucoma, retinal detachment, and macular degeneration. Current consensus defines myopia as a spherical equivalent (SE) refractive error of ≤−0.50 diopters (D). When SE ≤−3.00D, it is classified as mild myopia, and between −3.00D and >−6.00D as moderate myopia. SE≤−6.00D is classified as high myopia, usually accompanied by a significant increase in axial length (AL) (1). According to the World Health Organization, approximately 22% of the global population currently has some degree of myopia, a figure that is steadily rising. Epidemiological studies predict that by 2050, there will be 4.758 billion myopic individuals (49.8% of the world’s population) and 938 million highly myopic individuals (9.8% of the world’s population), severely impacting global vision health and quality of life (2, 3). Prevalence rates vary between countries, with East Asia and Southeast Asia reporting myopia rates as high as 80–90% among adults, and high myopia rates around 10–20% (4). Myopia not only affects individual learning, work, and daily life, but the increasing prevalence also indicates a rising burden of low vision and blindness due to pathological myopia, leading to significant socioeconomic costs (5).

Research on the treatment and management of myopia has been a focal point. Current clinical approaches to managing myopia progression include wearing orthokeratology lenses, defocus lenses, applying low-concentration atropine eye drops, repeated low-intensity red light therapy, and increasing outdoor activities. However, precision genetic treatment for myopia remains largely experimental in animals, and future efforts must explore multiple targets to develop treatments for this potentially blinding condition (6–8). In recent years, artificial intelligence and digital technologies have shown tremendous potential in myopia diagnosis and management, promising to provide advanced management tools in the future (9–14).

The exact mechanisms underlying the development and progression of myopia remain unclear, involving several signaling pathways such as TGF-*β*, cAMP, MMP-2, and hypoxia-inducible factor-1α (15). Previous studies have identified major risk factors for myopia, including prolonged near work, insufficient outdoor activities, high educational pressure, extended years of education, long screen time, and a family history of myopia (14, 16–20). Ocular parameters such as axial length (AL), corneal curvature, central corneal thickness (CCT), corneal radius of curvature (CRC), and axial length/corneal radius of curvature ratio (AL/CRC) are crucial factors in myopia development. Therefore, studying the relationships among these ocular parameters is vital for understanding the mechanisms of myopia, predicting its risk, and developing targeted prevention and intervention strategies (21–27).

This study aims to collect and analyze ocular parameter data from myopic patients to explore the differences in ocular parameters among patients with varying degrees of myopia and to investigate the correlation between axial length and other ocular parameters. Through these analyses, we hope to gain a more comprehensive understanding of the characteristics and mechanisms of myopia, providing a scientific basis for its prevention and treatment.

## 2 Materials and methods

### 2.1 Study design and participants

This study is a single-center observational study. Clinical data from adult myopic patients who visited the Eye Hospital of Nanjing Medical University from January to June 2023 were prospectively collected. Patients were screened according to predefined inclusion and exclusion criteria as follows:

Inclusion criteria:

1. Myopic patients aged between 18 and 45 years;
2. Best-corrected visual acuity (BCVA) ≥ 20/20;
3. Cessation of contact lens wear for more than 4 weeks;
4. Voluntary participation in relevant ophthalmic examinations, including visual acuity, intraocular pressure, slit-lamp examination, cycloplegic refraction, biometry measurements, and anterior segment panorama.

Exclusion criteria:

1. Presence of active inflammatory eye diseases;
2. Severe retinal pathology;
3. History of severe corneal disease;
4. History of ocular surgery;
5. History of severe ocular trauma.

The study protocol was approved by the Ethics Committee of Nanjing Medical University (Ethics No. (2021)-612).

### 2.2 Selection of study variables

The study collected and organized data on laterality, diopter of spherical power (DS), diopter of cylindrical power (DC), spherical equivalent (SE), axial length (AL, mm), central corneal thickness (CCT, μm), flat meridian keratometry (K1, D), steep meridian keratometry (K2, D), mean keratometry (Km, D), and anterior chamber depth (ACD, mm). Additionally, corneal radius of curvature (CRC) and axial length/corneal radius of curvature ratio (AL/CRC) were calculated. The extracted data were organized into an Excel file.

The formula for calculating the corneal radius of curvature is *K* = (n2–n1) * 1000/CRC, where K is the corneal curvature, CRC is the corneal radius of curvature, and n1 and n2 are the refractive indices of air and corneal aqueous humor, respectively. Assuming the refractive index of air is 1 and that of the corneal aqueous humor is 1.3375, the formula can be expressed as CRC = 337/Km.

AL/CRC is defined as AL divided by the average CRC measured at 90° and 180° meridians, i.e., AL/CRC = AL/CRC.

### 2.3 Grouping criteria

Patients were grouped based on the SE results of the examined eye: mild myopia (SE, ≤ −0.50D and > −3.00D), moderate myopia (SE, ≤ −3.00D and > −6.00D), and high myopia (SE, ≤ −6.00D).

Patients were also grouped based on the AL of the examined eye: short axial length (AL < 24 mm), medium axial length (24 mm ≤ AL < 26 mm), and long axial length (AL ≥ 26 mm).

### 2.4 Statistical analysis

Data were cleaned by removing outliers and imputing missing values using multiple imputation methods. Normality tests were performed on continuous variables. If the data followed a normal distribution, they were presented as mean ± standard deviation; otherwise, they were presented as median (P25, P75). Homogeneity of variance tests were conducted before comparing group differences. If homogeneity of variance was confirmed, ANOVA was used for comparison; otherwise, Welch ANOVA was used. Spearman correlation coefficients were calculated for continuous variables, and a correlation heatmap was drawn. Linear regression analysis and scatter plots were performed to assess the relationships between AL, AL/CRC, and SE. The accuracy of AL and AL/CRC in identifying high myopia was further evaluated by comparing the areas under the ROC curves (AUCs).

**Figure 1:**
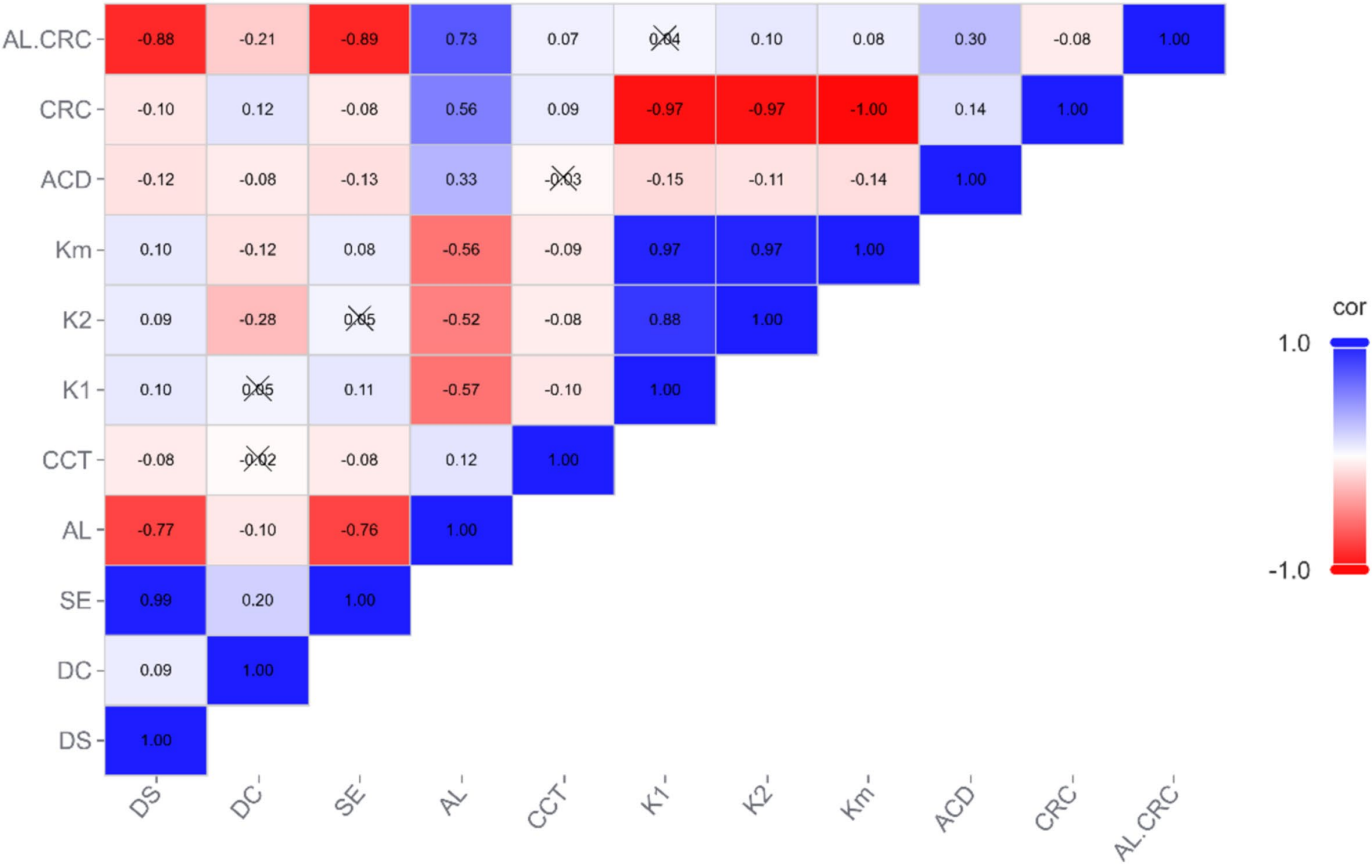
Heatmap of correlations between parameters.

**Figure 2:**
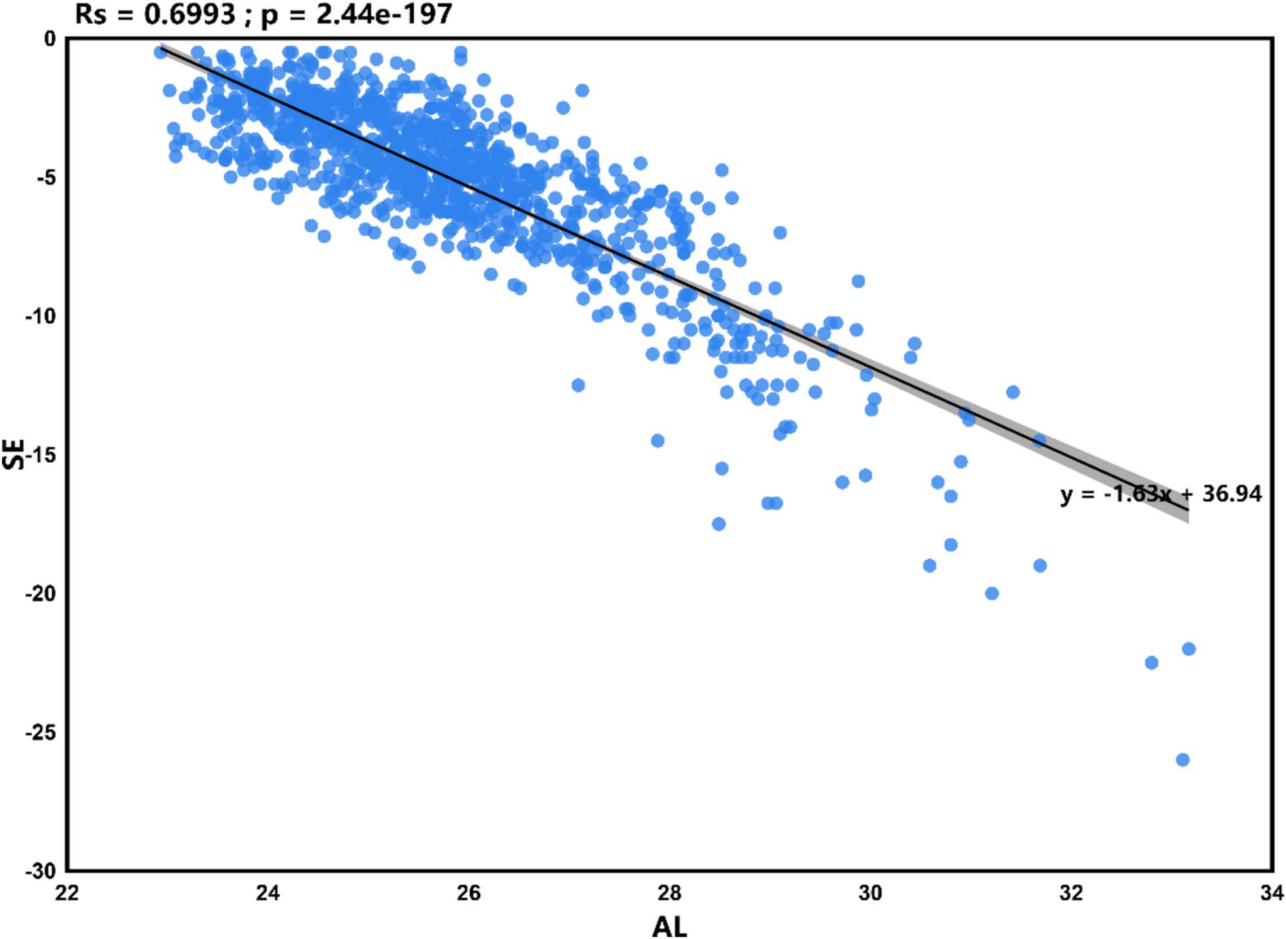
Scatter plot of linear regression between AL and SE.

**Figure 3:**
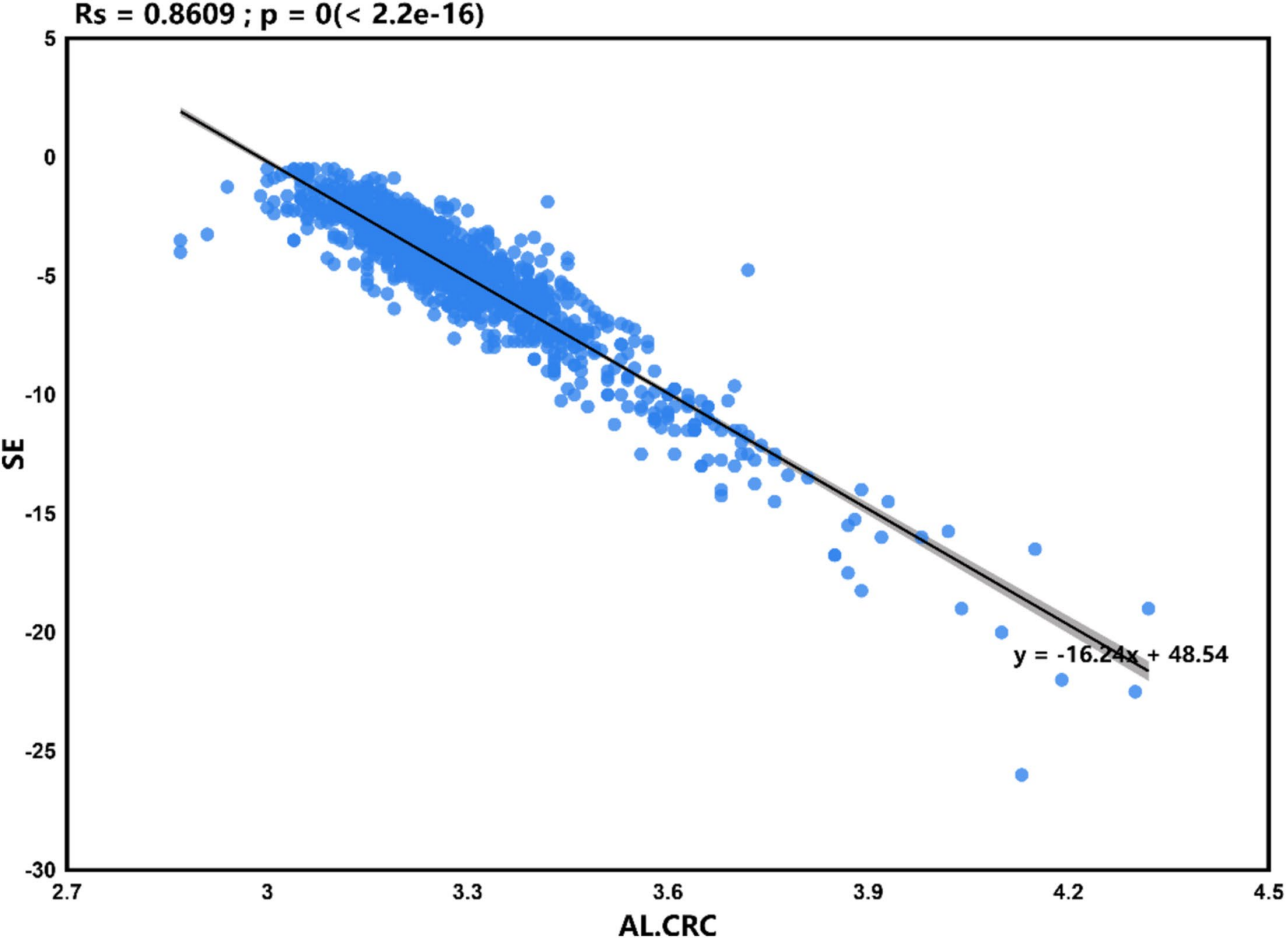
Scatter plot of linear regression between AL/CRC and SE.

**Figure 4:**
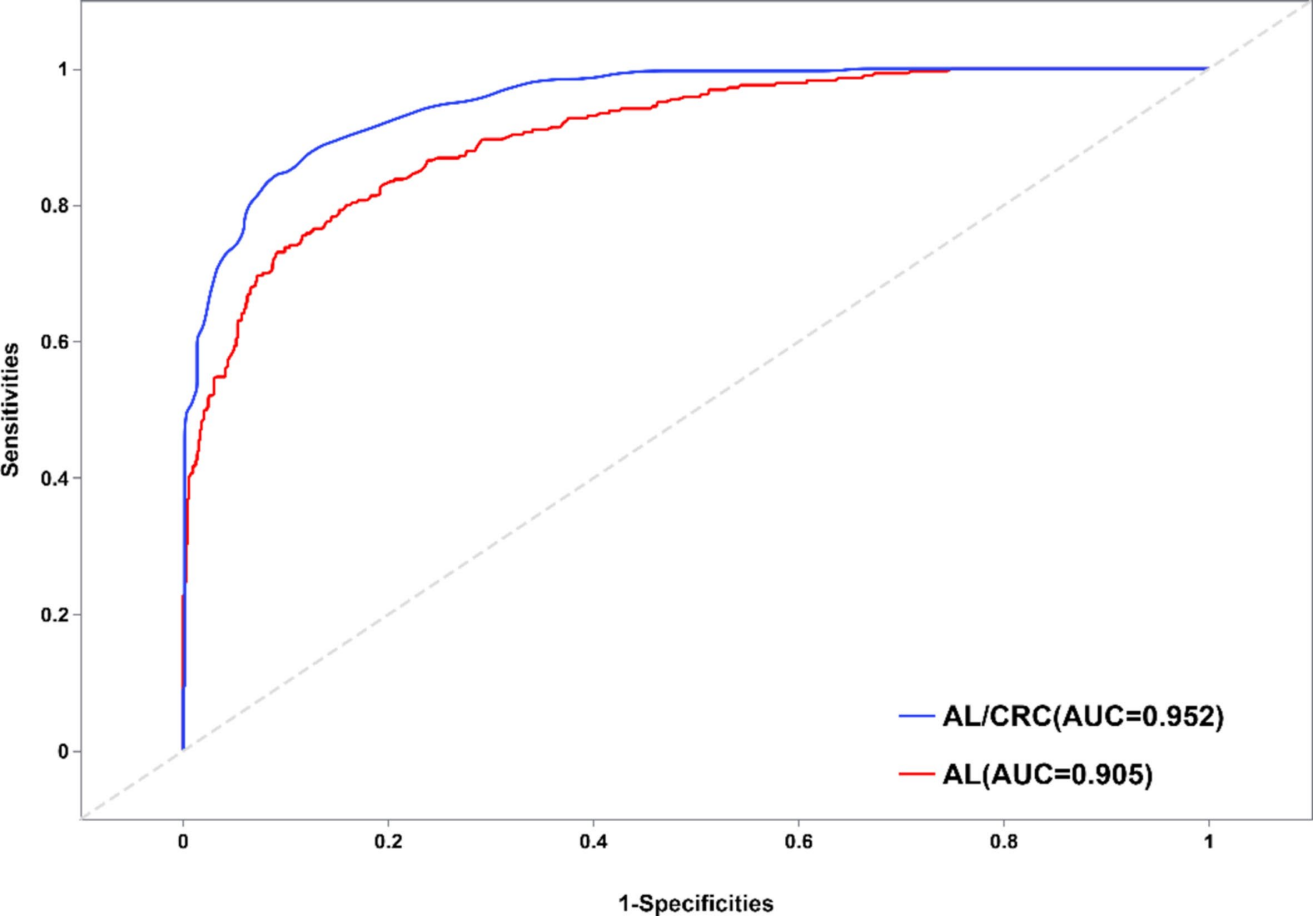
Schematic diagram of ROC curves for identifying high myopia with AL and AL/CRC parameters.

Statistical analysis was performed using SPSS version 26.0, with a significance level set at *p* < 0.05. The analysis results of Figures 1–4 plots were generated using the CNSknowall platform,^1^ a comprehensive web service for biomedical data analysis and visualization.

## 3 Results

### 3.1 Descriptive statistics of basic data

According to the predefined inclusion and exclusion criteria, a total of 537 myopic patients aged 18–45 years were initially enrolled in this study, comprising 1,074 eyes. However, due to refractive asymmetry in some patients, where one eye was myopic and the contralateral eye was either emmetropic or hyperopic, a total of 1,026 eyes were ultimately included for analysis. The basic data descriptive statistics are presented in Table 1. Based on the results in Table 1, it can be observed that the kurtosis of all parameters is <10 and skewness is <3, indicating a normal distribution of the study variables.

**Table 1:** Descriptive statistics of basic data.

Parameter	Unit	Min	Max	Mean	Standard deviation	Variance	Skewness	Kurtosis
DS	diopters (D)	−24.00	0.00	−4.7225	3.05811	9.352	−1.813	5.527
DC	diopters (D)	−4.00	0.00	−0.7902	0.67555	0.456	−1.096	1.484
SE	diopters (D)	−26.00	−0.50	−5.1176	3.12297	9.753	−1.836	5.693
AL	millimeters (mm)	22.93	33.17	25.8619	1.60576	2.578	0.974	1.367
CCT	micrometers (μm)	467.00	635.00	537.6316	29.28019	857.329	0.297	−0.019
K1	diopters (D)	36.50	47.30	42.4284	1.54020	2.372	−0.020	0.530
K2	diopters (D)	37.00	49.30	43.7802	1.67360	2.801	−0.062	0.446
Km	diopters (D)	36.80	48.00	43.0968	1.56838	2.460	−0.040	0.549
ACD	millimeters (mm)	2.47	4.01	3.1801	0.26114	0.068	−0.074	−0.099
CRC	–	7.02	9.16	7.8300	0.28685	0.082	0.330	0.897
AL/CRC	–	2.87	4.32	3.3036	0.17847	0.032	1.505	4.376

### 3.2 Differences among groups of different degrees of myopia

Data were grouped based on the SE results, with 242 eyes in the mild myopia group, 494 eyes in the moderate myopia group, and 290 eyes in the high myopia group. Fluctuations in data variability were examined using homogeneity of variance tests. The results indicated homogeneity of variance for CCT, K2, ACD, and CRC (*p* > 0.05), suggesting consistent data variability. Welch ANOVA was used for these variables. However, DS, DC, AL, K1, Km, and AL/CRC showed significant differences among groups (*p* < 0.05), indicating inconsistent data variability and the need for Welch ANOVA. Table 2 presents the statistical results of the differences among groups of different degrees of myopia, with significant differences observed in eight parameters among the groups (*p* < 0.05).

**Table 2:** Statistical analysis of differences among groups of different degrees of myopia.

Parameter	Unit	Group	Mean	Standard deviation	F/Welch F	Significance, *p*-value
DS	diopters (D)	Mild Myopia	−1.6839	0.68646	960.850a	0.000***
		Moderate Myopia	−4.0810	0.87536		
		High Myopia	−8.3509	3.13415		
DC	diopters (D)	Mild Myopia	−0.6550	0.58840	29.471a	0.000***
		Moderate Myopia	−0.7105	0.60211		
		High Myopia	−1.0388	0.78948		
AL	millimeters (mm)	Mild Myopia	24.5290	0.77210	509.563a	0.000***
		Moderate Myopia	25.5209	0.98674		
		High Myopia	27.5552	1.55666		
CCT	micrometers (μm)	Mild Myopia	534.4793	28.13559	2.286b	0.103
		Moderate Myopia	537.9615	29.96293		
		High Myopia	539.7000	28.91827		
K1	diopters (D)	Mild Myopia	42.5281	1.38143	7.777a	0.000***
		Moderate Myopia	42.5554	1.63555		
		High Myopia	42.1288	1.46043		
K2	diopters (D)	Mild Myopia	43.8224	1.51496	2.233b	0.108
		Moderate Myopia	43.8603	1.77673		
		High Myopia	43.6086	1.61033		
Km	diopters (D)	Mild Myopia	43.1669	1.39777	4.722a	0.009**
		Moderate Myopia	43.2020	1.67820		
		High Myopia	42.8590	1.48688		
ACD	millimeters (mm)	Mild Myopia	3.1610	0.24028	10.127b	0.000***
		Moderate Myopia	3.1563	0.27405		
		High Myopia	3.2365	0.24727		
CRC	–	Mild Myopia	7.8152	0.25762	4.687b	0.010*
		Moderate Myopia	7.8124	0.30507		
		High Myopia	7.8725	0.27424		
AL/CRC	–	Mild Myopia	3.1395	0.06854	667.863a	0.000***
		Moderate Myopia	3.2678	0.08330		
		High Myopia	3.5014	0.18233		

The “F/Welch F” column includes the “a” and “b” labels to indicate the homogeneity of variance for each parameter. “a” denotes parameters for which homogeneity of variance was confirmed, and a standard ANOVA was used. “b” indicates parameters with non-homogeneous variance, for which Welch ANOVA was applied. *p < 0.05, **p < 0.01, ***p < 0.001.

### 3.3 Differences among groups of different axial lengths

Data were grouped based on the axial length (AL) results, with 111 eyes in the short axial length group, 522 eyes in the medium axial length group, and 393 eyes in the long axial length group. Similar to the previous analysis, homogeneity of variance tests was conducted, with consistent data variability observed for CCT, K1, K2, Km, and ACD (*p* > 0.05). Welch ANOVA was used for these variables. However, DS, DC, SE, CRC, and AL/CRC showed significant differences among groups (*p* < 0.05), indicating inconsistent data variability and the need for Welch ANOVA. Table 3 presents the statistical results of the differences among groups of different axial lengths, with significant differences observed in ten parameters among the groups (*p* < 0.05).

**Table 3:** Statistical analysis of differences among groups of different axial lengths.

Parameter	Unit	Group	Mean	Standard deviation	F/Welch F	Significance, *p*-value
DS	diopters (D)	Short AL	−2.1396	1.22413	338.314a	0.000***
		Medium AL	−3.4684	1.49494		
		Long AL	−7.1177	3.36567		
DC	diopters (D)	Short AL	−0.8131	0.61373	12.973a	0.000***
		Medium AL	−0.6901	0.60360		
		Long AL	−0.9167	0.75754		
SE	diopters (D)	Short AL	−2.5462	1.19480	340.763a	0.000***
		Medium AL	−3.8135	1.52247		
		Long AL	−7.5760	3.43863		
CCT	micrometers (μm)	Short AL	527.8919	26.88100	9.444b	0.000***
		Medium AL	537.4119	29.64474		
		Long AL	540.6743	28.89214		
K1	diopters (D)	Short AL	44.1387	1.32933	187.181b	0.000***
		Medium AL	42.7280	1.25830		
		Long AL	41.5473	1.36979		
K2	diopters (D)	Short AL	45.6351	1.39053	162.044b	0.000***
		Medium AL	44.0207	1.39098		
		Long AL	42.9369	1.56379		
Km	diopters (D)	Short AL	44.9000	1.30175	188.657b	0.000***
		Medium AL	43.3626	1.27958		
		Long AL	42.2344	1.42116		
ACD	millimeters (mm)	Short AL	3.0228	0.22916	56.693b	0.000***
		Medium AL	3.1456	0.25274		
		Long AL	3.2703	0.24885		
CRC	–	Short AL	7.5118	0.21546	186.081a	0.000***
		Medium AL	7.7784	0.22934		
		Long AL	7.9885	0.27355		
AL/CRC	–	Short AL	3.1537	0.08898	318.504a	0.000***
		Medium AL	3.2323	0.09219		
		Long AL	3.4406	0.19444		

The “F/Welch F” column includes the “a” and “b” labels to indicate the homogeneity of variance for each parameter. “a” denotes parameters for which homogeneity of variance was confirmed, and a standard ANOVA was used. “b” indicates parameters with non-homogeneous variance, for which Welch ANOVA was applied. *p < 0.05, **p < 0.01, ***p < 0.001.

### 3.4 Correlation analysis between parameters

Spearman correlation coefficients were calculated among the variables, and a matrix heatmap was plotted to visualize these correlations. In the heatmap, each cell’s color represents the magnitude of the correlation between the corresponding variables. Blue indicates a positive correlation, while red indicates a negative correlation. The intensity of the colors varies according to the correlation coefficients: darker shades represent stronger correlations, while lighter shades indicate weaker correlations. Variables with absolute correlation coefficients >0.7 were considered to have strong correlations.

The correlation analysis in this study revealed strong relationships between AL, SE, DS, and AL/CRC. Specifically, the strongest correlations were observed between AL and SE, as well as AL and DS. These strong positive correlations align with clinical knowledge, suggesting that changes in axial length are closely linked with variations in SE and refractive status. The AL/CRC parameter also demonstrated strong correlations with DS and SE, highlighting its potential as a composite measure for assessing refractive errors and supporting clinical diagnoses and prognostic evaluations.

In contrast, we noted weaker correlations between some of the other parameters, indicating that they are less directly related to the primary measures of axial length and refractive error. These weaker correlations may reflect secondary factors that are less influential in determining refractive status.

### 3.5 Linear regression analysis between AL and SE

Based on the results in Figure 1 of section 3.4, this study found that the correlation coefficient between AL and SE is −0.78, suggesting a strong correlation between them. Setting SE as the dependent variable, the linear regression analysis between AL and SE showed a determination coefficient *R*^2^ = 0.699, indicating that the independent variable (AL) can explain 69.9% of the variance in the dependent variable (SE). The statistical test results of the model using analysis of variance (ANOVA) showed *F* = 2382.166 (*p* = 0.000), indicating the model’s statistical significance. Further calculation of the model’s intercept, regression coefficient of the independent variable, 95% confidence interval (CI), *t*-value, and *p*-value showed that the intercept of the regression model is 36.94, and the non-standardized coefficient (i.e., slope) of the independent variable (AL) is −1.63 (95% CI, −1.69 to −1.56). This indicates that for every 1 mm increase in AL, SE decreases by −1.63D, as illustrated in Figure 2. Based on this, the regression equation for this case can be written as:


$$SE=36.94+\left(-1.63\times AL\right)$$

This equation can be used to calculate the corresponding SE within a reasonable range of AL.

### 3.6 Linear regression analysis between AL/CRC and SE

According to the results in Figure 1 of section 3.4, this study found that the correlation coefficient between AL/CRC and SE is −0.89, indicating a strong correlation between them. Setting SE as the dependent variable, the linear regression analysis between AL/CRC and SE showed a determination coefficient *R*^2^ = 0.861, indicating that the independent variable (AL/CRC) can explain 86.1% of the variance in the dependent variable (SE). The statistical test results of the model using analysis of variance (ANOVA) showed *F* = 6358.316 (*p* = 0.000), indicating the model’s statistical significance. Further calculation of the model’s intercept, regression coefficient of the independent variable, 95% confidence interval (CI), *t*-value, and *p*-value showed that the intercept of the regression model is 48.54, and the non-standardized coefficient of the independent variable (AL/CRC) is −16.24 (95% CI: −16.64 to −15.84). This indicates that for every 1-unit increase in AL/CRC, SE decreases by −16.24D, as illustrated in Figure 3. Based on this, the regression equation for this case can be written as:


$$SE=48.54+\left(-16.24\times AL/CRC\right)$$

This equation can be used to calculate the corresponding SE within a reasonable range of AL/CRC.

### 3.7 Role of AL and AL/CRC parameters in identifying high myopia

Based on the SE results of patients, AL and AL/CRC parameters were separately set as independent variables, and whether they had high myopia as the dependent variable. By comparing the area under the receiver operating characteristic (ROC) curves of the two indicators, the differences in the identification accuracy of high myopia between the two indicators were explored. The ROC curve is a visual tool for evaluating the classification method. When the AUC > 0.9, it indicates very high accuracy of the indicator, which can better distinguish between cases and non-cases. The results of this study found that the AUC for predicting high myopia through AL was 0.905 (95% CI: 0.885–0.925), and through AL/CRC was 0.952 (95% CI: 0.940–0.965). Both indicators showed high diagnostic accuracy, as shown in Figure 4. Furthermore, the statistical test results comparing the areas under the two indicators’ curves (AL-AL/CRC) showed a *Z*-value of −4.947, *p*-value of 0.000, and a difference in AUC of −0.048, indicating a statistically significant difference in the areas under the curves of the two diagnostic methods. Thus, it can be seen that the accuracy of AL/CRC in identifying high myopia is superior to that of AL, and the difference is statistically significant (*p* < 0.05).

## 4 Discussion

This study comprehensively collected and analyzed ocular parameters in adult myopic patients to explore the differences in biological measurement indicators such as AL, corneal curvature, CCT, CRC, and AL/CRC among patients with different degrees of myopia. Furthermore, it investigated the correlation between axial length, AL/CRC, and the SE of myopic patients.

Firstly, significant differences were found in ocular parameters among patients with different degrees of myopia. Specifically, parameters such as AL, corneal curvature (K1, K2, Km), and ACD showed significant differences among patients with different degrees of myopia. In particular, AL significantly increased with the degree of myopia, while corneal curvature and anterior chamber depth showed varying trends. In our study, we found that CCT did not exhibit statistically significant differences across different myopia severity groups, which is consistent with the findings of several previous studies (28–31). This supports the notion that CCT may not be closely associated with the degree of myopia. However, we acknowledge that other studies have reported significant correlations between CCT and myopia severity, with some suggesting that CCT might play a role in myopic defocus compensation (24, 25). One possible explanation for this discrepancy could be the differences in study populations, methodologies, or sample sizes. For example, certain studies may have focused on populations with more extreme forms of myopia or employed different techniques for measuring CCT, which could lead to different results. Additionally, genetic and environmental factors could contribute to variations in CCT that may not be fully captured in our study.

Secondly, significant differences were observed in various ocular parameters among patients with different axial lengths. Specifically, as the axial length increased, CCT, ACD, CRC, and AL/CRC all showed a gradual increase. It is worth noting that in this study, CCT increased gradually with the increase in AL. However, a study by Jin et al. found a continuous decrease in CCT with the elongation of AL (25). Additionally, a retrospective multicenter study revealed a negative correlation between CCT and the rate of AL elongation, suggesting that thin CCT may be associated with accelerated myopia progression (32). Therefore, further evidence from evidence-based medicine is needed to explore the correlation between CCT and the degree of myopia and axial length.

Finally, this study focused on exploring the correlation between AL, AL/CRC, and SE of myopic patients and predicting high myopia. The results showed that compared to AL, AL/CRC had a stronger correlation with SE and better accuracy in identifying high myopia. Consistent with previous studies, most research on AL/CRC has focused on preschool children. Since preschool children cannot cooperate with cycloplegic refraction, some studies suggest that the AL/CRC ratio can be used as an alternative indicator to identify preschool children with low myopic reserves and myopia, aiding clinicians and parents in timely screening for low myopic reserve children before primary school (33, 34). Similarly, Fan et al.’s study also showed that the correlation between AL/CRC and SE was greater than that between AL and SE in adult myopic patients, suggesting that the AL/CRC index can to some extent be independent of refractive analysis and suitable for diagnosing high myopia in adults (35).

To further evaluate the diagnostic value of AL and AL/CRC for high myopia, this study employed ROC curve analysis. The results showed that the AUC for predicting high myopia was 0.905 (95% CI: 0.885–0.925) through AL and 0.952 (95% CI: 0.940–0.965) through AL/CRC, with a statistically significant difference in the areas under the curves of the two diagnostic methods. This indicates that AL/CRC significantly outperforms AL in identifying high myopia, demonstrating its superior predictive ability for high myopia.

AL/CRC measurement has several advantages. Firstly, it provides a reliable alternative method for diagnosing myopia in patients who cannot undergo cycloplegic refraction, especially in preschool children and patients with angle-closure glaucoma (26). Secondly, AL/CRC better reflects the biomechanical performance and morphological pathological changes caused by myopia progression, making it a more reliable indicator for diagnosing myopia and assessing the risk of fundus lesions in highly myopic patients (36). Thirdly, AL/CRC can also reflect the situation of peripheral retinal defocus. Patients with larger AL/CRC values are more likely to have myopic defocus around the retina, leading to further elongation of the axial length (27, 35, 36). However, while AL/CRC can be used to classify myopia of different grades, its potential for monitoring disease progression remains uncertain. Further evidence is needed to assess whether AL/CRC can accurately track changes in the degree of myopia over time and monitor the progression of myopia-related complications. Some studies have found that the progression of AL/CRC does not strongly correlate with the progression of SE, indicating that the relationship between AL/CRC and myopia progression is still not fully understood (37). This highlights the need for further investigation into the dynamic use of AL/CRC in clinical settings, particularly to determine its role in longitudinal monitoring of myopia and related ocular conditions.

This study has several limitations that should be addressed in future research. First, the small sample size may affect the generalizability of the findings, and larger studies are needed to improve statistical power. Additionally, the data were collected from a single medical institution, which may limit the external validity of the results. A multi-center approach could provide more representative data. The six-month observation period may not be sufficient to capture long-term myopia progression, and longer follow-up is needed. Moreover, while this study explored the relationship between the AL/CRC ratio and SE, it did not consider potential confounding factors such as age, gender, ethnicity, and other physiological characteristics (e.g., ocular diameter, lens thickness), which may influence the applicability of the AL/CRC ratio. Future studies should include more diverse populations and investigate how these factors impact the performance of the AL/CRC ratio to enhance its clinical relevance.

This study offers preliminary insights into the relationships among various ocular indicators of myopia. The results of the study indicate significant differences in eight indicators among patients with different degrees of myopia and ten indicators among patients with different axial lengths. There is a significant negative correlation between AL, AL/CRC, and SE. Compared to the AL index, the AL/CRC index has a stronger correlation with SE and better accuracy in identifying high myopia. The AL/CRC index measurement holds promise as an accurate and convenient alternative method for refractive examination.

## 5 Conclusion

This study analyzed ocular biological indicators in myopic patients, revealing significant variations across degrees of myopia and axial lengths. The AL/CRC index demonstrated a stronger correlation with SE and greater accuracy in identifying high myopia compared to the AL index, highlighting its potential as a precise and convenient alternative for refractive examination, particularly in patients unable to undergo cycloplegic refraction. These findings contribute valuable insights into the assessment of myopia and suggest promising directions for refining diagnostic methods in clinical practice.

## Data Availability

The raw data supporting the conclusions of this article will be made available by the authors, without undue reservation.

## Footnotes

^1^ https://cnsknowall.com/#/HomePage
